# Apocrine Mixed Tumor of the Upper Cutaneous Lip: A Case Report

**DOI:** 10.7759/cureus.78623

**Published:** 2025-02-06

**Authors:** Alyssa Francesca Ahorro, Jack Huang, Christopher Weyer, Jamie M Moenster

**Affiliations:** 1 School of Osteopathic Medicine, University of the Incarnate Word, San Antonio, USA; 2 Department of Pathology, Tucson Pathology Associates, Tucson, USA; 3 Department of Dermatology and Plastic Surgery, Dermatology and Plastic Surgery of Arizona, Tucson, USA; 4 Department of Plastic Surgery, Dermatology and Plastic Surgery of Arizona, Tucson, USA

**Keywords:** apocrine mixed tumor, chondroid syringoma, chondromyxoid stroma, cutaneous adnexal neoplasm, mohs surgery

## Abstract

Chondroid syringomas are rare adnexal neoplasms composed of epithelial and mesenchymal components, posing diagnostic challenges due to their diverse histological features. We report an atypical presentation of an apocrine mixed tumor in a 51-year-old female patient who presented with a 0.7 cm firm, flesh-colored nodule on the right lateral cutaneous lip. The patient’s clinical findings included significant actinic damage, such as dyschromia, elastosis, lentigines, and actinic keratoses, indicative of chronic sun exposure. Over four months, the lesion increased in size to 1.7 cm x 1.2 cm, warranting surgical excision. Histopathological analysis revealed a cutaneous adnexal neoplasm with follicular and apocrine differentiation, consistent with a chondroid syringoma. The tumor exhibited the characteristic chondromyxoid stroma with areas of myxoid and chondroid components.

Mohs micrographic surgery was performed, ensuring complete tumor removal while sparing healthy tissue. A rotational flap closure provided an excellent esthetic and functional outcome, with minimal scarring and no functional impairments reported during follow-up.

This case highlights the diagnostic and therapeutic challenges associated with chondroid syringomas, particularly in atypical locations such as the cutaneous lip. The coexistence of chronic photodamage further complicates the clinical picture, emphasizing the importance of thorough histopathological evaluation. Despite the tumor's rarity, timely surgical intervention and meticulous wound management yielded a favorable prognosis. This report underscores the need for clinician awareness to facilitate early diagnosis and appropriate management of rare cutaneous neoplasms, with follow-up care recommended to monitor for potential recurrence.

## Introduction

Chondroid syringomas are rare adnexal neoplasms that contain mesenchymal and epithelial components, which are believed to derive from the ductal and secretory segments of the sweat gland (apocrine and eccrine) [1]. These tumors are characterized as mixed tumors due to their histological presentation demonstrating a mix of epithelial, apocrine, and myoepithelial cells embedded within a stromal matrix that can include myxoid, chondroid, or osseus elements [2]. These tumors exhibit diverse cellular components and stroma, making accurate histopathological examination essential for diagnosis.

Histologically, chondroid syringomas are well-circumscribed, lobulated masses, often encapsulated by a fibrous pseudocapsule. Microscopically, they show broad sheets of squamous epithelial cells, sometimes with intracellular bridges, embedded within a connective tissue stroma that frequently exhibits varying degrees of cartilaginous differentiation. Glandular spaces in these tumors demonstrate marked variability in size and shape, typically lined by either flattened cuboidal cells or a distinctive double epithelial layer, the latter being a critical diagnostic feature of apocrine mixed tumors. Additional areas of squamous and osseous metaplasia may also be observed, adding further complexity to the tumor's structure [3]. The encapsulated, lobulated nature of chondroid syringomas, coupled with their diverse cellular composition, is essential for distinguishing them from other cutaneous neoplasms [4].

While these tumors are rare, it is critical to recognize and differentiate from other cutaneous neoplasms. The rarity of apocrine mixed tumors, especially in atypical locations, and their varied histological presentation pose a diagnostic challenge for clinicians. Understanding the typical features of these tumors aids in differentiating them from other skin neoplasms, leading to appropriate management and treatment.

## Case presentation

A 51-year-old female patient with no significant past medical or family history presented to dermatology with concerns regarding a mass located on her right lateral cutaneous lip at the vermillion border. Her initial evaluation revealed a raised, firm, flesh-colored nodule clinically measured at approximately 0.7 cm. Additional examination findings included extensive actinic damage on the entire face, with notable dyschromia and elastosis in sun-exposed areas, multiple lentigines, and actinic keratoses. These findings were indicative of chronic photodamage and aging, commonly seen in patients with prolonged sun exposure.

**Figure 1:**
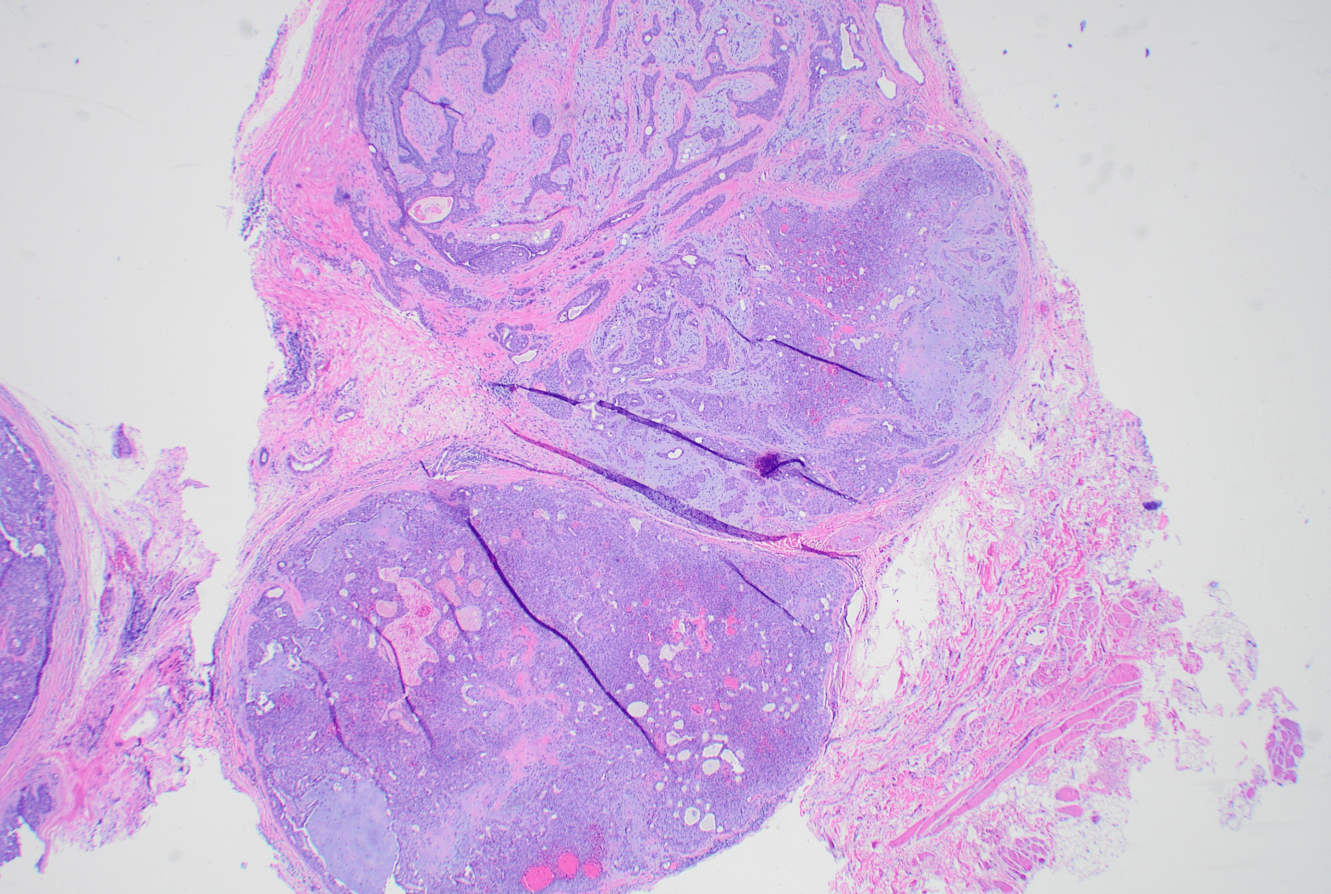
A low-power view of this tumor shows well-circumscribed and focally encapsulated lobular proliferation of both mixed epithelial and stromal components. The epithelial cells are interconnected sheets of squamoid cells with duct formation. The intermingled stromal part appears as myxoid and chondroid.

**Figure 2:**
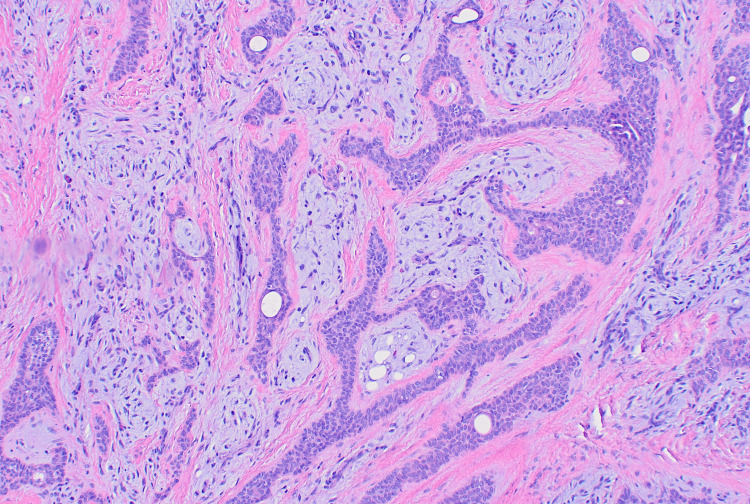
A higher-power view of this tumor shows squamoid epithelial cells focally form duct structures and the adjacent stroma is myxoid.

Chondroid syringomas are typically known as slow-growing masses, but in this case, the tumor did not follow this usual course. At a follow-up visit four months later, the mass on the patient’s right upper cutaneous lip had notably increased in size, measuring 1.7 cm x 1.2 cm. Given the tumor's size and location, as well as the patient's extensive sun exposure, it was decided to proceed with surgical excision. Histopathological examination confirmed the diagnosis of a cutaneous adnexal neoplasm with follicular and apocrine differentiation, consistent with an apocrine mixed tumor. The tumor exhibited the characteristic chondromyxoid stroma, further supporting the diagnosis of chondroid syringoma. Histological images (Figures 1, 2) revealed the well-circumscribed lobular proliferation of mixed epithelial and stromal components, with areas of myxoid and chondroid stroma interspersed with squamoid epithelial cells and ductal formation. Given the pathology, secondary excision with Mohs micrographic surgery (MMS) for frozen section (FS) analysis was performed. Although MMS is typically reserved for malignant tumors, it was utilized in this benign case to ensure complete tumor removal while preserving normal tissue in a cosmetically sensitive area.

**Figure 3:**
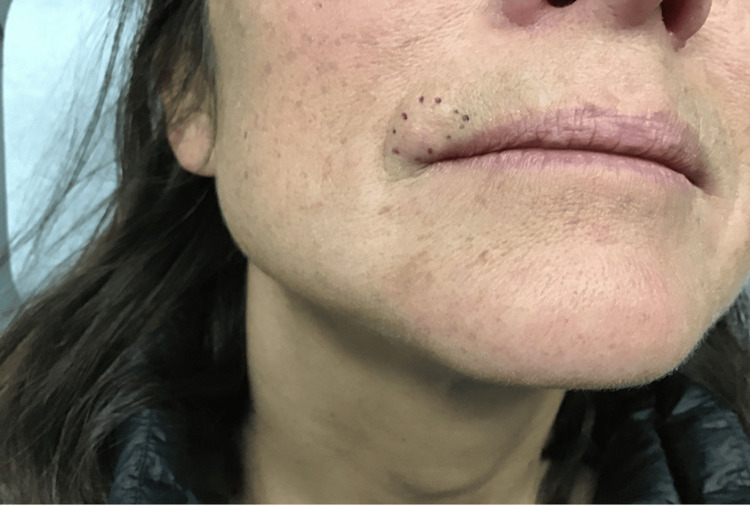
Preoperative photograph of the patient’s right upper cutaneous lip, with the mass clearly outlined at the vermillion border. The lesion, measuring approximately 1.7 cm x 1.2 cm, demonstrates a raised, firm, and flesh-colored appearance, consistent with the clinical presentation of an apocrine mixed tumor. This image highlights the precise localization of the tumor prior to surgical excision and reconstruction.

**Figure 4:**
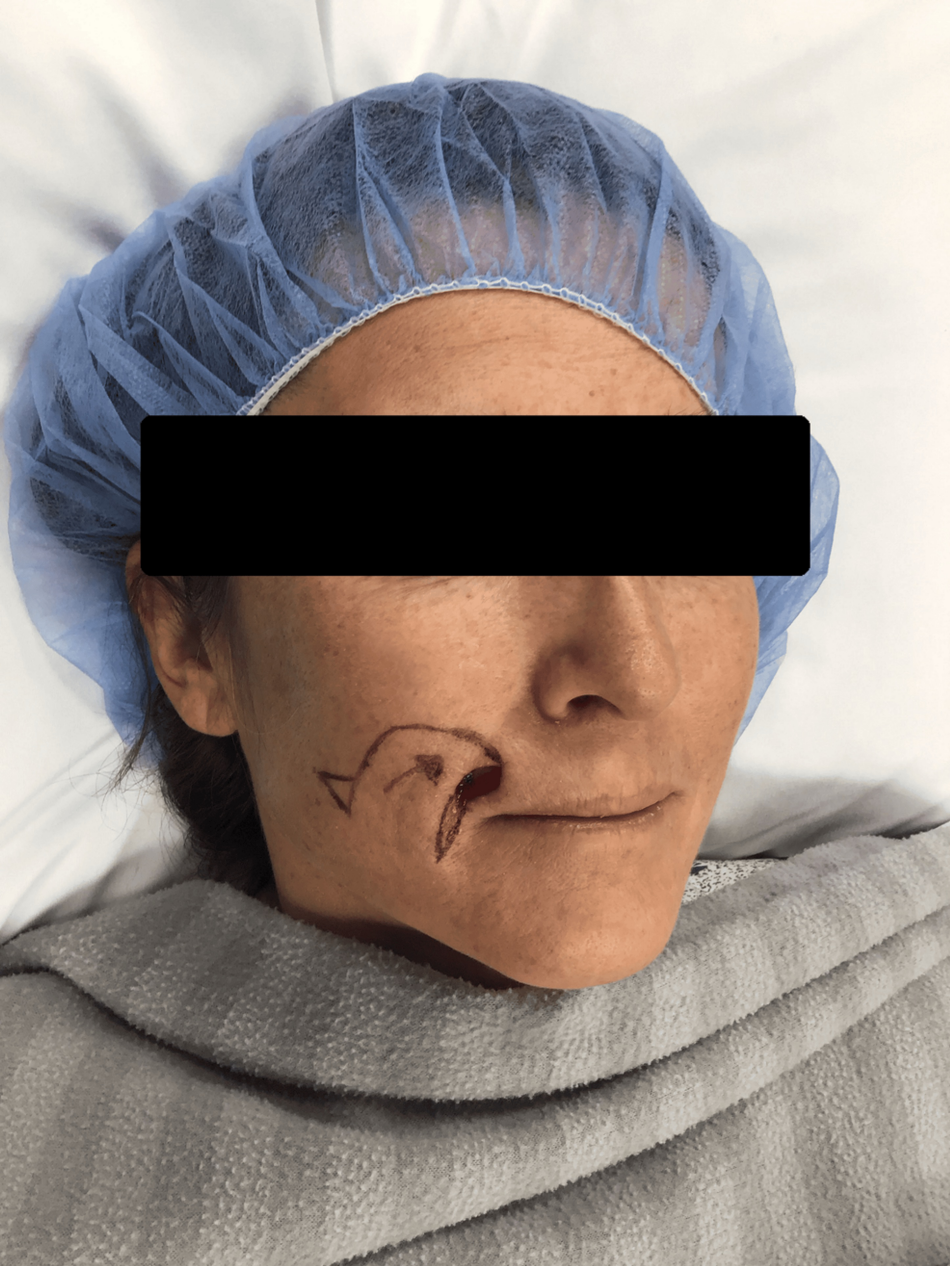
Intraoperative image following complete surgical resection. The well-defined defect demonstrates the extent of tissue excision necessary to achieve clear margins while preserving surrounding healthy tissue.

**Figure 5:**
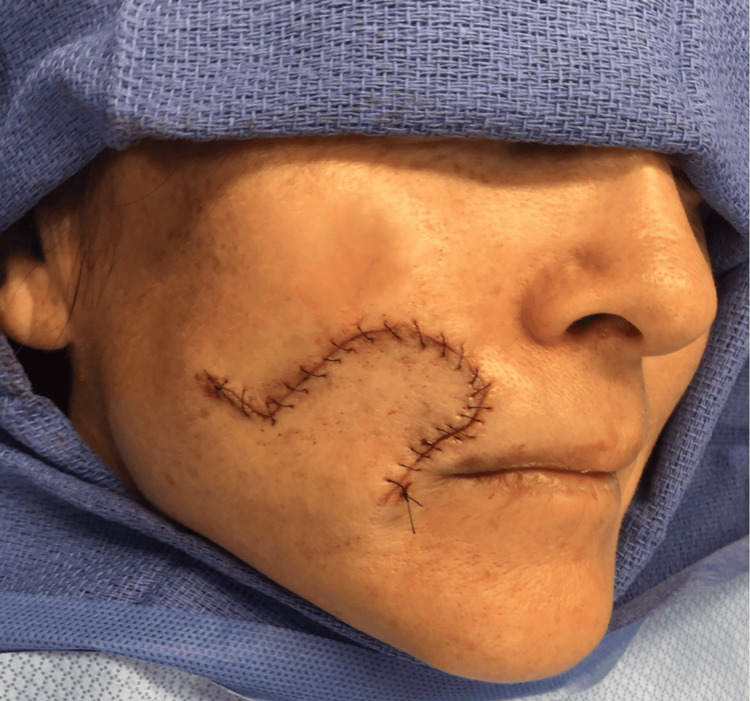
Postoperative photograph taken on the day of closure, illustrating the repair of the patient's right upper cutaneous lip with a rotation flap technique aimed at achieving an optimal esthetic outcome for the lip and facial contours.

**Figure 6:**
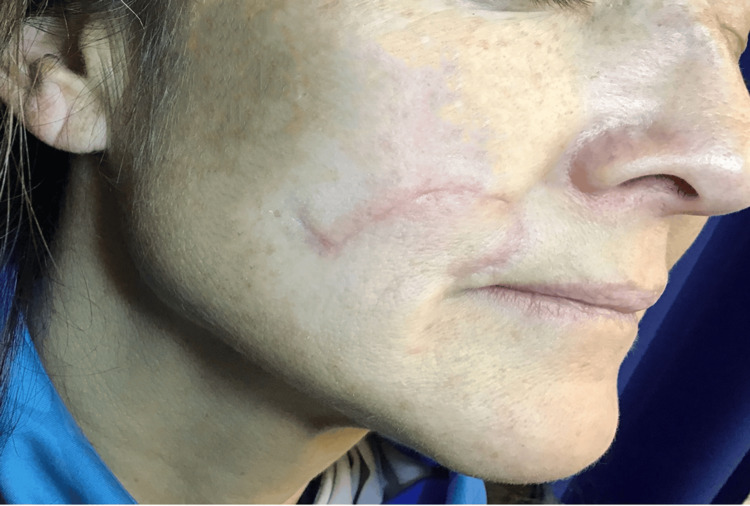
Three months postoperative photograph of the patient’s right upper cutaneous lip, demonstrating excellent healing following surgical excision of the apocrine mixed tumor and closure with a rotational flap. The site exhibits minimal scarring, well-integrated tissue contours, and no evidence of functional impairment.

The Mohs excision removed the 1.5 cm x 1.2 cm mass from the right upper cutaneous lip. A rotational flap closure was subsequently performed to preserve a good esthetic outcome for the lip and face (Figures 3-5). The patient demonstrated optimal wound healing, with minimal scarring observed at follow-up visits (Figure 6). No functional impairments were reported, and the patient has continued to adhere to the prescribed wound care regimen.

Given the benign nature of apocrine mixed tumors and the complete excision of the lesion, recurrence is unlikely. The patient will continue to be monitored through routine follow-ups to ensure there are no signs of recurrence, though these tumors are known to have an excellent prognosis after surgical removal.

## Discussion

This case presents several noteworthy aspects. First, the location of the apocrine mixed tumor on the right upper cutaneous lip at the vermillion border is highly unusual. Apocrine mixed tumors are most commonly found in areas with a higher concentration of apocrine glands, such as the axilla and anogenital regions [5]. While rare, previous case reports have documented occurrences of apocrine mixed tumors in atypical sites, including the head and neck region, highlighting the diverse anatomical presentations of these neoplasms [6,7].

Second, the patient exhibited significant actinic damage, including dyschromia, elastosis, lentigines, and actinic keratoses, features commonly associated with chronic sun exposure and aging. The presence of extensive actinic damage posed a diagnostic challenge, as actinic keratosis is a known precursor to squamous cell carcinoma [8]. Similar cases in the literature have emphasized the difficulty of distinguishing benign adnexal neoplasms from malignant cutaneous lesions in sun-damaged skin, underscoring the necessity of histopathological evaluation for definitive diagnosis [9].

Third, the histopathological diagnosis of a cutaneous adnexal neoplasm with apocrine differentiation, specifically an apocrine mixed tumor, is relatively rare, with limited cases reported in the literature [10]. Additionally, while MMS is typically reserved for malignant tumors, it was utilized in this benign case to ensure complete tumor removal while preserving as much normal tissue as possible in a cosmetically sensitive area. This approach is particularly relevant in the head and neck region, where tissue conservation is critical for optimal reconstructive outcomes.

Finally, the patient’s excellent postoperative outcome, with minimal scarring and no functional restrictions following excision and rotational flap closure, reinforces the effectiveness of surgical management in cosmetically sensitive areas. This case underscores the importance of timely diagnosis and appropriate surgical intervention in managing rare cutaneous tumors, particularly in patients with extensive sun exposure.

## Conclusions

In conclusion, this case underscores the rarity and diagnostic complexity of apocrine mixed tumors, particularly when they present in atypical locations, such as the cutaneous lip. Despite the unusual site of involvement, the patient experienced a favorable postoperative course, highlighting the importance of timely surgical intervention and meticulous wound management. Given the benign nature of these tumors and the low risk of recurrence following complete excision, the prognosis remains excellent. However, this case emphasizes the need for heightened awareness among clinicians, to facilitate early recognition and accurate diagnosis of such rare cutaneous neoplasms. Future recommendations include continued awareness through routine follow-up care, despite the infrequent recurrence of apocrine mixed tumors, to ensure early detection of potential reoccurrence.
